# Impacts of Two Land-Use Conversion Pathways on Soil Properties and Microbial Communities in Tropical Forests of Hainan Island

**DOI:** 10.3390/plants15203091

**Published:** 2026-10-09

**Authors:** Guihua Wang, Yubo Li, Fengying Han, Changxi Wang, Qi Yu, Beibei Zhang

**Affiliations:** 1 College of Forestry Engineering, Shandong Agriculture and Engineering University, Jinan 250100, China; 2 School of Tropical Agriculture and Forestry, Hainan University, Danzhou 571737, China; 3 Hainan Seed Industry Laboratory, Sanya 572024, China

**Keywords:** land-use conversion, microbial communities, conversion pathways, tropical forest, soil physicochemical properties, high-throughput sequencing

## Abstract

Large-scale tropical forest conversion to plantations or cropland severely impacts soil health. However, most studies have relied on pairwise designs, single-type chronosequences, or multi-type comparisons that are often confounded by environmental heterogeneity, thereby overlooking the complexity of successive land-use transitions on a common land-use background. Here, we investigated the responses of soil physicochemical properties and microbial communities to two conversion pathways (NSF → ORF → YRF; NSF → ORF → URF → AL) within a single farm, where shared soil parent material and climate allowed for a direct comparison of land-use types with different conversion pathways. Soil properties and microbial diversity were analyzed using physicochemical methods and high-throughput sequencing, with samples collected from five land-use types (six biological replicates per type). Results showed NSF had the highest soil properties. Along NSF → ORF → YRF, TOC, DOC, TN, IN and C/N declined gradually. In contrast, URF exhibited severe degradation, with AL showing partial recovery. Bacterial diversity remained relatively stable, whereas fungal communities were highly sensitive, with diversity decreasing progressively and dominant phyla shifting with intensified disturbance. DOC, EC and SM were most strongly associated with microbial community ordination, with pH showing no significant association. URF had the highest predicted abundance of C/N/S cycling genes, while NSF maintained a balanced fungal trophic structure. This study provides a comparative assessment of soil properties and microbial communities along two tropical land-use conversion pathways, contributing empirical evidence for sustainable land-use management in tropical regions.

## 1. Introduction

Tropical forests are vital components of terrestrial ecosystems, boasting extremely high biodiversity and efficient material cycling functions [1]. They play an irreplaceable role in providing key ecosystem services, such as maintaining global carbon sinks, regulating climate, conserving water and soil, and preserving biodiversity [1,2,3]. However, driven by the rising demand for land to support human activities, tropical land use patterns have undergone progressive and multi-stage transformation processes rather than single pairwise conversions. Typically, taking tropical forests as the original type, forestland is first converted into plantations [4,5]. Once these plantations mature and yields decline, or when profit incentives shift, they are subsequently either replanted or replaced with other crops [6,7,8], ultimately forming a successive land-use conversion chain. This successive land-use transitions not only alters surface vegetation coverage and community structure, but also exerts a cumulative impact on the health and functional stability of soil ecosystems [9,10,11].

Soil microorganisms, as the core functional carriers of soil ecosystems, dominate key ecological processes such as organic matter decomposition and nutrient cycling, with their activities being modulated by soil physicochemical properties [12,13,14]. The dynamic changes in both microorganisms and physicochemical properties serve as sensitive indicators of soil ecosystem health [15,16]. Currently, studies on tropical land-use conversion in the context of soil properties and microbial communities can be broadly categorized into three main types. The first and most prevalent type is binary comparisons between a single land-use type and its natural forest reference, such as natural forest versus rubber plantation [17,18]. The second type examines land-use types of different ages within the same land-use category [19,20]. The third type employs spatial juxtapositions to compare multiple land-use types within a unified framework [21,22]. However, even in this third approach, the sites examined may vary in soil parent material, topography, or climate, which complicates the attribution of observed differences to land-use type alone. Moreover, these studies rarely consider successive land-use transition pathways originating from the same initial land-use type. Stage-specific responses to environmental gradients have been documented in other systems, such as plants along soil water gradients [23]. Nevertheless, whether soil physicochemical properties and microbial communities exhibit analogous stage-specific dynamics along successive land-use conversion pathways remains largely unknown.

Among the various land-use conversion types, abandoned land represents a critical endpoint and an important window for observing ecosystem responses. Globally, secondary succession on abandoned agricultural lands has been shown to drive substantial changes in soil properties and microbial communities, with most studies reporting overall recovery trends [24,25,26]. In tropical regions, natural restoration on abandoned lands can alter nutrient cycling and microbial community composition over time, although recovery patterns are modulated by factors such as parent material and prior land-use intensity [27,28,29]. However, most of these studies have focused on relatively long-term succession, and the earliest stages of abandonment remain poorly understood. In Hainan, several studies have examined soil carbon dynamics following land-use conversion [30,31]. Soil organic carbon fractions were reported to follow the order paddy field > abandoned cropland > vegetable land, with paddy soils showing higher carbon sequestration potential [30], while carbon mineralization was found to be highest in abandoned land, associated with elevated dissolved organic carbon and copiotrophic microbial taxa [31]. Nevertheless, these studies have primarily focused on carbon-related properties, with limited attention given to the integrated responses of soil physicochemical properties and microbial communities to cropland abandonment.

In China, Hainan Island is the largest tropical island and is considered a global biodiversity hotspot [32]. Here, land-use conversion characterized by rubber plantation expansion and agricultural intensification is typical of tropical successive land-use changes driven by socioeconomic factors [33]. Specifically, at the Liangyuan Experimental Agricultural Farm, the adjacent distribution of land-use types, including natural secondary forest, old rubber forest, young rubber forest, cropland, and abandoned land, along with well-documented management histories, minimizes interference from background edaphic and climatic factors. This provides an ideal natural experimental site for exploring the effects of successive land-use conversions for tropical soil ecosystems.

Therefore, this study selected Liangyuan Experimental Agricultural Farm in Nada Town, Danzhou City, Hainan Island as the research site, and integrated soil physicochemical analysis and high-throughput sequencing technology to focus on the two typical tropical successive land-use transition pathways: NSF → ORF → YRF (natural secondary forest → old rubber forest → young rubber forest) and NSF → ORF → URF →AL (natural secondary forest → old rubber forest → young rubber forest → upland cropland → abandoned land). The objectives were to: (1) characterize the stage-specific changes in soil physicochemical properties and microbial community composition along the successive land-use conversion; (2) identify the physicochemical factors significantly associated with microbial community variation across the land-use conversion sequence; and (3) assess the early-stage responses of soil physicochemical properties and microbial communities in abandoned land (AL) following cropland abandonment. This study addresses a critical research gap in tropical soil microbial ecology by examining successive land-use transition, enhances our understanding of soil–vegetation–microorganism interactions under progressive anthropogenic disturbance, and provides stage-specific evidence to inform tropical soil conservation, restoration of degraded ecosystems, and sustainable land-use management.

## 2. Results

### 2.1. Soil Physicochemical Properties Under Two Land-Use Conversion Pathways

The physicochemical properties of soil exhibited stage-dependent trends along the two land-use conversion pathways (Table 1). Specifically, NSF had the highest contents of TOC (14.24 ± 1.81 g/kg), DOC (0.231 ± 0.008 g/kg), TN (505.30 ± 43.06 mg/kg), and IN (7.60 ± 0.60 mg/kg), as well as a moderate C/N ratio (28.11 ± 1.17). Along the NSF → ORF → YRF pathway, TOC, TN, and SM declined gradually and continously, with TOC decreasing from 14.24 ± 1.81 g/kg to 7.45 ± 0.20 g/kg, TN from 505.30 ± 43.06 mg/kg to 351.15 ± 2.95 mg/kg, and SM from 20.03 ± 3.78% to 19.08 ± 1.78%. Along the NSF → ORF → URF pathway, URF showed the most severe degradation among all land-use types, with the lowest TOC (4.35 ± 0.57 g/kg), TN (281.82 ± 11.80 mg/kg), and SM (12.45 ± 0.52%), and the highest EC (294.43 ± 21.00 μS/cm). In addition, soil BD in ORF (1.45 ± 0.07 g/cm^3^) and YRF (1.47 ± 0.09 g/cm^3^) was significantly higher than that in NSF (1.22 ± 0.06 g/cm^3^) and URF (1.23 ± 0.06 g/cm^3^), while AL showed intermediate values for most physicochemical indicators, falling between the degraded agricultural site (URF) and the forested/reforested sites (NSF, ORF, YRF).

### 2.2. Soil Microbial Community Composition Under Two Land-Use Conversion Pathways

**Figure 1:**
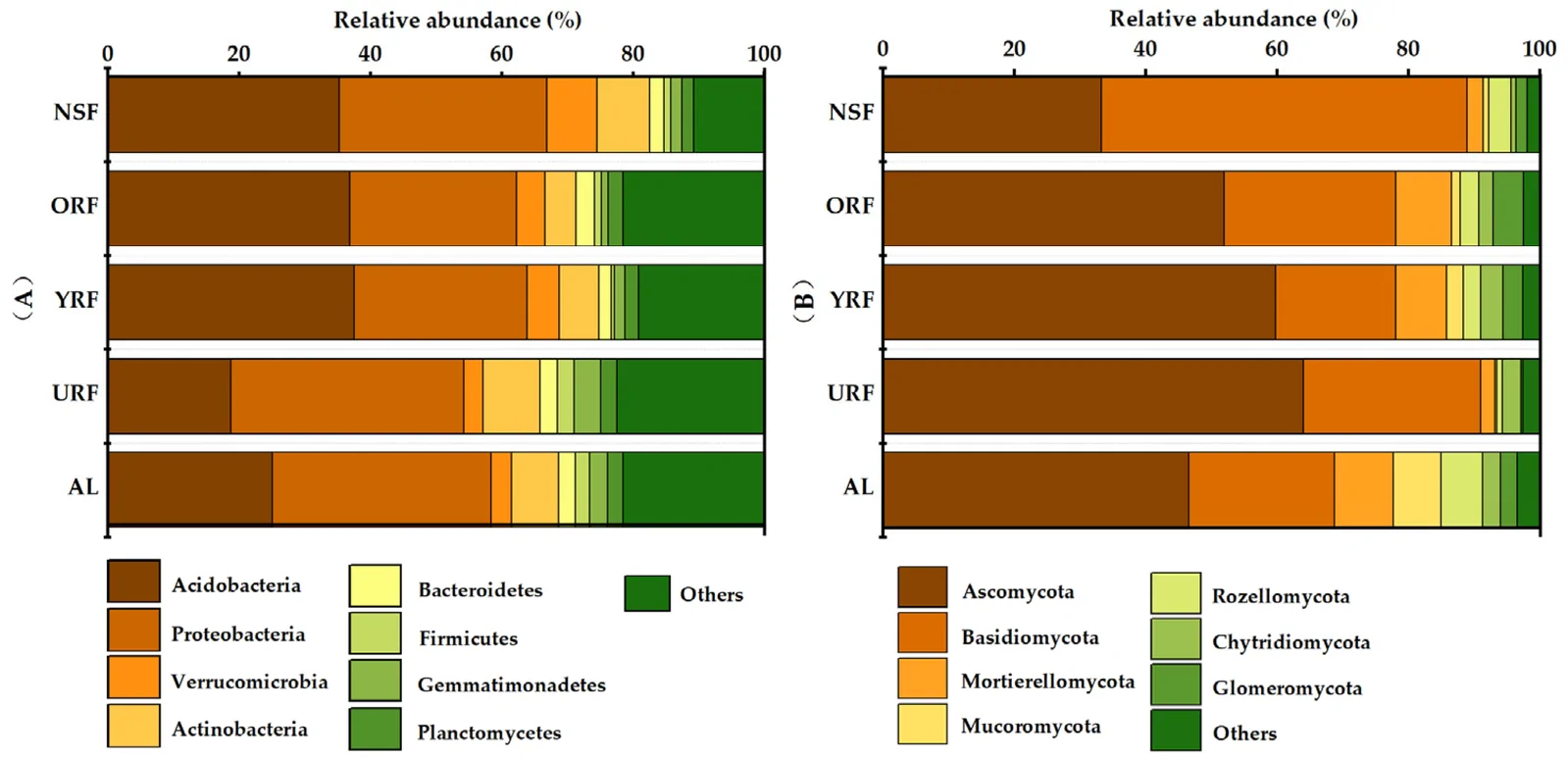
Relative abundance of soil bacterial communities (**A**) and fungal communities (**B**) at the phylum level. NSF, Natural Secondary Forest; ORF, Old Rubber Forest; YRF, Young Rubber Forest; URF, Upland Rice Field; AL, Abandoned Land. Only phyla with relative abundance > 1% in at least one sample are shown.

A total of 16 bacterial phyla and 15 fungal phyla were identified in the five land-use types (Tables S1 and S2). At the phylum level (relative abundance > 1%), the dominant taxonomic composition of soil microorganisms was consistent, but their relative abundances differed significantly. For bacterial communities (Figure 1A), all five land-use types were dominated by Acidobacteria, Proteobacteria, Verrucomicrobia, Actinobacteria, Bacteroidetes, Firmicutes, Gemmatimonadetes, and Planctomycetes. The combined relative abundance of the top two dominant phyla, namely Acidobacteria and Proteobacteria, ranged from 54.20% to 66.87%. Along the NSF → ORF → YRF pathway, Acidobacteria was the most dominant phylum in NSF, ORF, and YRF, with relative abundances ranging from 35.30% to 37.50%. In contrast, Proteobacteria was the dominant phylum in URF and AL along the NSF → ORF → URF → AL pathway. Additionally, Verrucomicrobia in NSF and Actinobacteria in NSF, AL, and URF all exhibited relative abundances exceeding 7%. For fungal communities (Figure 1B), the dominant phyla across all land-use types included Ascomycota, Basidiomycota, Mortierellomycota, Mucoromycota, Rozellomycota, Chytridiomycota, and Glomeromycota. The combined relative abundance of Ascomycota and Basidiomycota ranged from 68.78% to 91.01%, representing the core dominant phyla. Ascomycota was the most dominant phylum in ORF and YRF (NSF → ORF → YRF pathway) as well as URF (NSF → ORF → URF → AL pathway), with relative abundances of 51.97–63.99%. In contrast, Basidiomycota dominated NSF, accounting for 55.65% of the total fungal community. Notably, in AL, the relative abundances of Mortierellomycota (8.89%), Mucoromycota (7.32%), and Rozellomycota (6.27%) all exceeded 5%, which were significantly higher than those in other land-use types.

**Figure 2:**
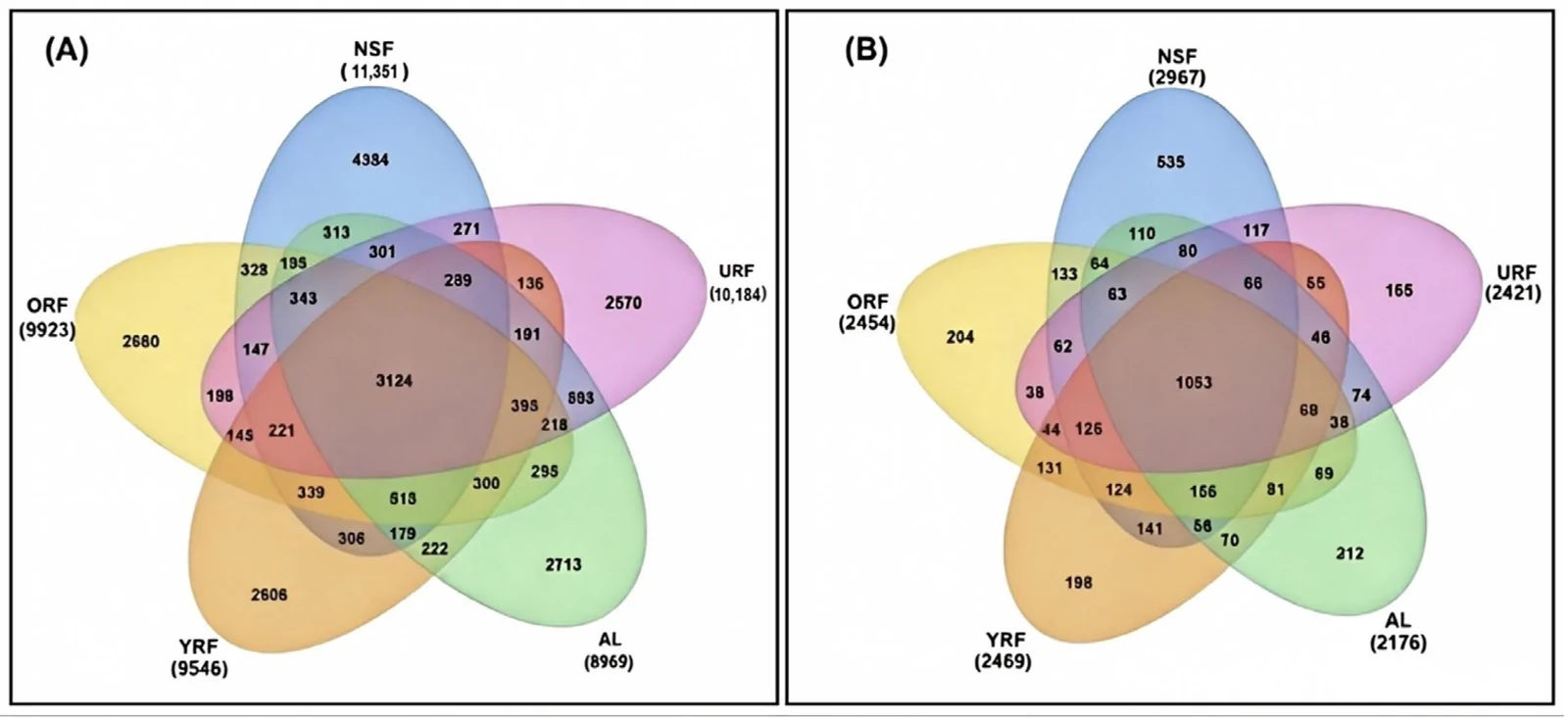
Venn diagram analysis of soil bacterial (**A**) and fungal (**B**) communities in five land use types. NSF, Natural Secondary Forest; ORF, Old Rubber Forest; YRF, Young Rubber Forest; URF, Upland Rice Field; AL, Abandoned Land. Numbers indicate OTUs at a 97% similarity threshold.

The Venn diagram shows OTU distribution across two land-use conversion pathways (Figure 2). Along the NSF → ORF → YRF pathway, total bacterial OTUs gradually decreased from 11,351 in NSF to 9923 in ORF and 9546 in YRF (Figure 2A). Fungal OTUs decreased from 2967 in NSF to 2454 in ORF, then slightly increased to 2469 in YRF (Figure 2B). The proportion of unique bacterial OTUs dropped from 39% in NSF to 27% in both ORF and YRF, while unique fungal OTUs decreased from 18% in NSF to 8% in ORF and YRF. Notably, ORF shared 5010 bacterial and 1781 fungal OTUs with NSF, while YRF shared 4869 bacterial and 1803 fungal OTUs with NSF. Along the NSF → ORF → URF →AL pathway, total bacterial OTUs displayed a pattern of decrease – increase – decrease: 11,351 (NSF) → 9923 (ORF) → 10,184 (URF) → 8969 (AL). In contrast, total fungal OTUs continuously declined, reaching 2176 in AL (the lowest among all land-use types). The proportion of unique bacterial OTUs decreased from 39% in NSF to 27% in ORF and 25% in URF, then rebounded to 30% in AL. For fungal OTUs, the proportion of unique taxa decreased from 18% in NSF to 8% in ORF and 7% in URF, increasing to 14% in AL. Importantly, ORF exhibited greater OTU overlap with NSF than did URF, which shared 4794 bacterial and 1648 fungal OTUs with NSF. Similarly, AL shared 5057 bacterial and 1663 fungal OTUs with NSF, also showing higher OTU overlap with NSF than URF.

### 2.3. Soil Microbial Diversity Under Two Land-Use Conversion Pathways

**Figure 3:**
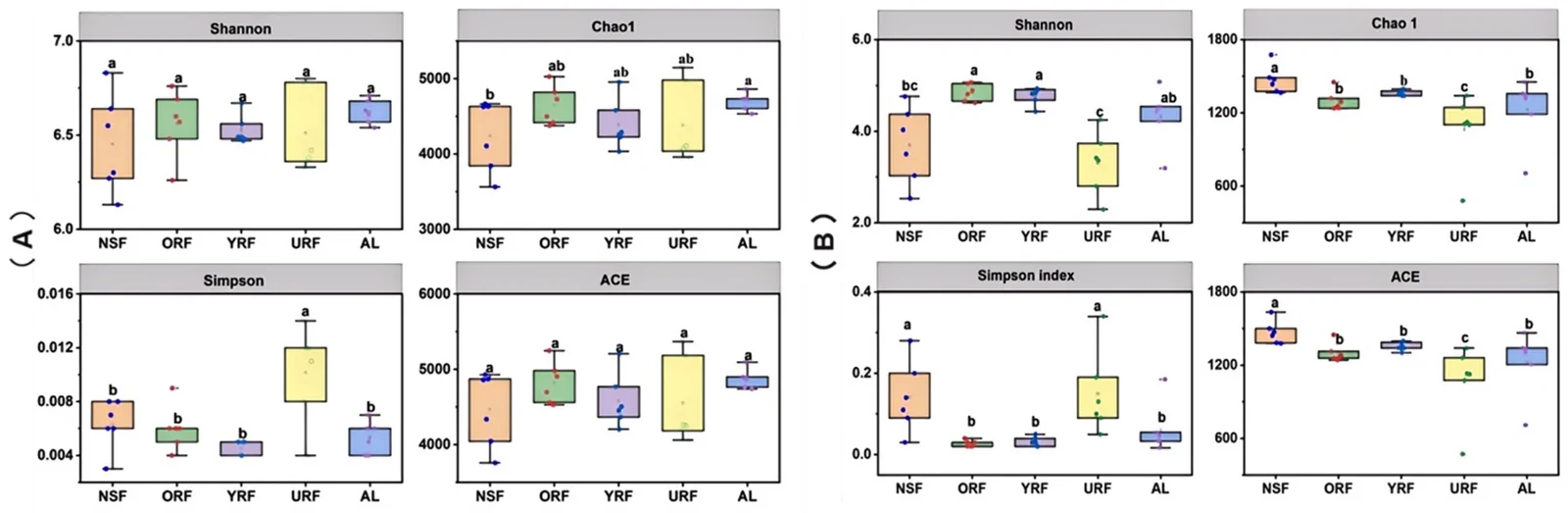
Alpha-diversity indices of soil bacteria (**A**) and fungi (**B**) among five land use types. NSF, Natural Secondary Forest; ORF, Old Rubber Forest; YRF, Young Rubber Forest; URF, Upland Rice Field; AL, Abandoned Land. Different lowercase letters indicate significant differences among land use types (*p* < 0.05, Duncan’s test). Values are presented as mean ± standard deviation (SD).

Along the NSF → ORF → YRF pathway (Figure 3), no significant differences in the Shannon index were detected among NSF, ORF, and YRF for bacterial communities. The Simpson index remained stable, while the Chao1 and ACE indices only exhibited marginal fluctuations without significant changes. For fungal communities, the Shannon index of NSF was significantly lower than that of ORF and YRF. Conversely, the Simpson index of NSF was significantly higher than that of ORF and YRF, and both the Chao1 and ACE indices decreased significantly from NSF to ORF and YRF. Along the NSF → ORF → URF → AL pathway (Figure 3A), bacterial communities showed a significantly higher Simpson index compared to NSF, with no significant changes in the Chao1 and ACE indices. After URF was abandoned to form AL, the Chao1 index of AL was significantly higher than that of NSF, while the other indexes were similar to that of NSF. For fungal communities, from NSF to URF, the Shannon index first increased and then decreased, whereas the Simpson index first decreased and then increased, and the Chao1 and ACE indices were at their lowest levels (Figure 3B). Following the abandonment of URF to AL, the Shannon index of AL was significantly higher than that of URF, and the Chao1 and ACE indices were comparable to those observed in ORF and YRF, but remained significantly lower than those of NSF and higher than those of URF.

**Figure 4:**
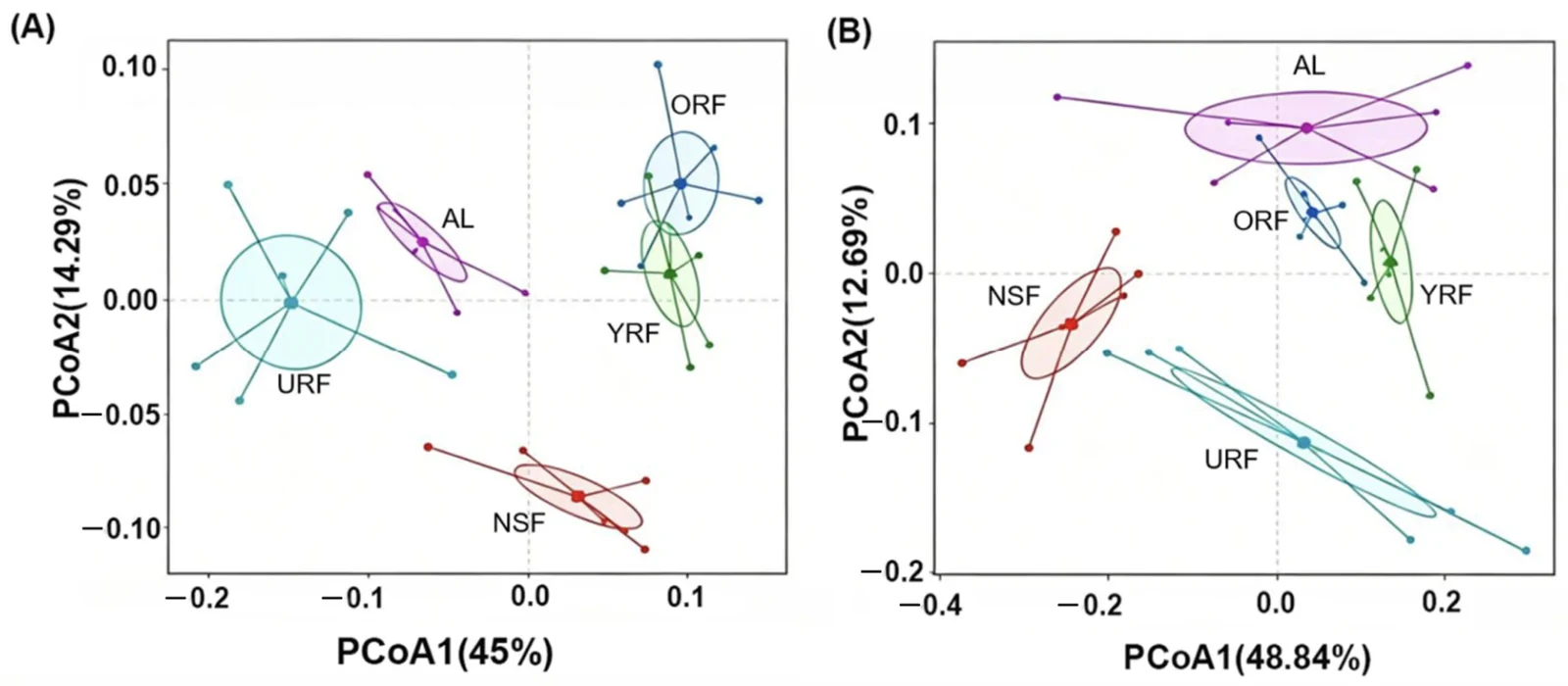
Principal Coordinate Analysis (PCoA) of Bray–Curtis distance from bacterial (**A**) and fungal (**B**) communities based on OTU levels. NSF, Natural Secondary Forest; ORF, Old Rubber Forest; YRF, Young Rubber Forest; URF, Upland Rice Field; AL, Abandoned Land. Percentages in axis labels indicate variation explained by each principal coordinate.

Principal Coordinate Analysis (PCoA) based on Bray–Curtis distance revealed distinct community structures among the five land-use types (Figure 4A,B). For bacterial communities, the first principal component (PCoA1) explained 45% of the total variation, while the second principal component (PCoA2) explained 14.29%. PERMANOVA confirmed significant differences in bacterial community composition (R^2^ = 0.6026, *p* < 0.001). Along the plantation pathway, ORF and YRF clustered closely together, indicating high similarity in their bacterial community structures. Similarly, AL and URF along the agricultural pathway showed high similarity. In contrast, the bacterial community of NSF was clearly separated from the other four land-use types. For fungal communities, PCoA1 explained 48.84% of the total variation and PCoA2 explained 12.69%. PERMANOVA revealed significant community differentiation (R^2^ = 0.5862, *p* < 0.001). The fungal communities of NSF and URF formed distinct, separate clusters, while ORF and YRF (the NSF → ORF → YRF pathway) clustered closely with AL (the NSF → ORF → URF → AL pathway). In summary, the agricultural pathway (NSF → ORF → URF → AL) induced more pronounced community shifts than the rubber plantation pathway (NSF → ORF → YRF), with the ORF → URF transition identified as the critical stage where both bacterial and fungal communities showed the greatest divergence from the forest reference.

### 2.4. Relationship Between Soil Microbial Communities and Soil Properties

**Figure 5:**
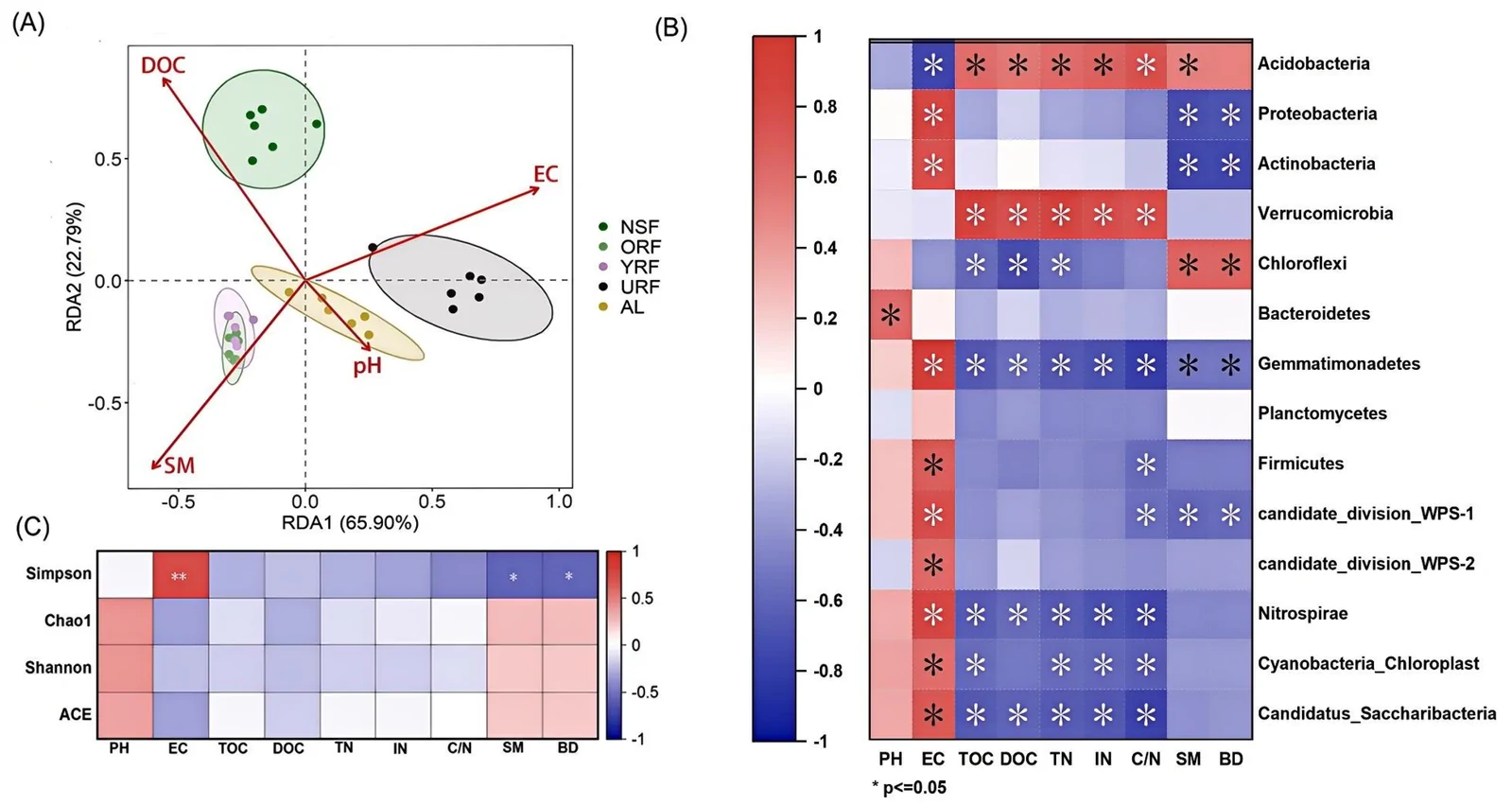
Relationship between soil physicochemical properties and soil bacterial community composition and diversity indices. (**A**) Redundancy analysis (RDA) ordination of bacterial communities constrained by soil properties. (**B**) Pearson correlation heatmap between dominant bacterial phyla (relative abundance > 1%) and soil properties. (**C**) Pearson correlation heatmap between bacterial diversity indices and soil properties. NSF, Natural Secondary Forest; ORF, Old Rubber Forest; YRF, Young Rubber Forest; URF, Upland Rice Field; AL, Abandoned Land. * *p* < 0.05, ** *p* < 0.01. The color scale in panels (**B**,**C**) represents Spearman’s correlation coefficients, with red indicating positive correlations and blue indicating negative correlations.

Redundancy analysis (RDA) examined relationships between soil bacterial communities and physicochemical properties across five land-use types. Results showed that the global RDA model was statistically significant (F = 4.046, *p* = 0.001). The retained soil physicochemical variables collectively explained 88.69% of total variance in soil bacterial community composition, with RDA1 and RDA2 explaining 65.90% and 22.79%, respectively (Figure 5A). Marginal tests of the RDA model indicated that only DOC showed a significant marginal effect on bacterial community composition (*p* = 0.026), whereas pH (P = 0.581), EC (*p* = 0.129), and SM (*p* = 0.221) were non-significant. Envfit vector fitting indicated DOC (R^2^ = 0.864, *p* < 0.001), SM (R^2^ = 0.819, *p* < 0.001) and EC (R^2^ = 0.818, *p* < 0.001) were strongly correlated with the RDA ordination space, while pH showed weak and non-significant correlation (R^2^ = 0.125, *p* = 0.145). Pearson correlation analysis revealed variable relationships between bacterial phyla and soil properties (Figure 5B). Of the 14 main bacterial phyla, 11 showed significant correlations with soil physicochemical properties. For example, soil TOC, DOC, TN, IN, and C/N were positively correlated with dominant phyla including Acidobacteria and Verrucomicrobia, while negatively correlated with Gemmatimonadetes, Nitrospirae, and Candidatus_Saccharibacteria (*p* < 0.05). In addition, soil properties were also correlated with bacterial diversity. The Simpson index was negatively correlated with soil SM and BD, but positively correlated with EC (Figure 5C).

**Figure 6:**
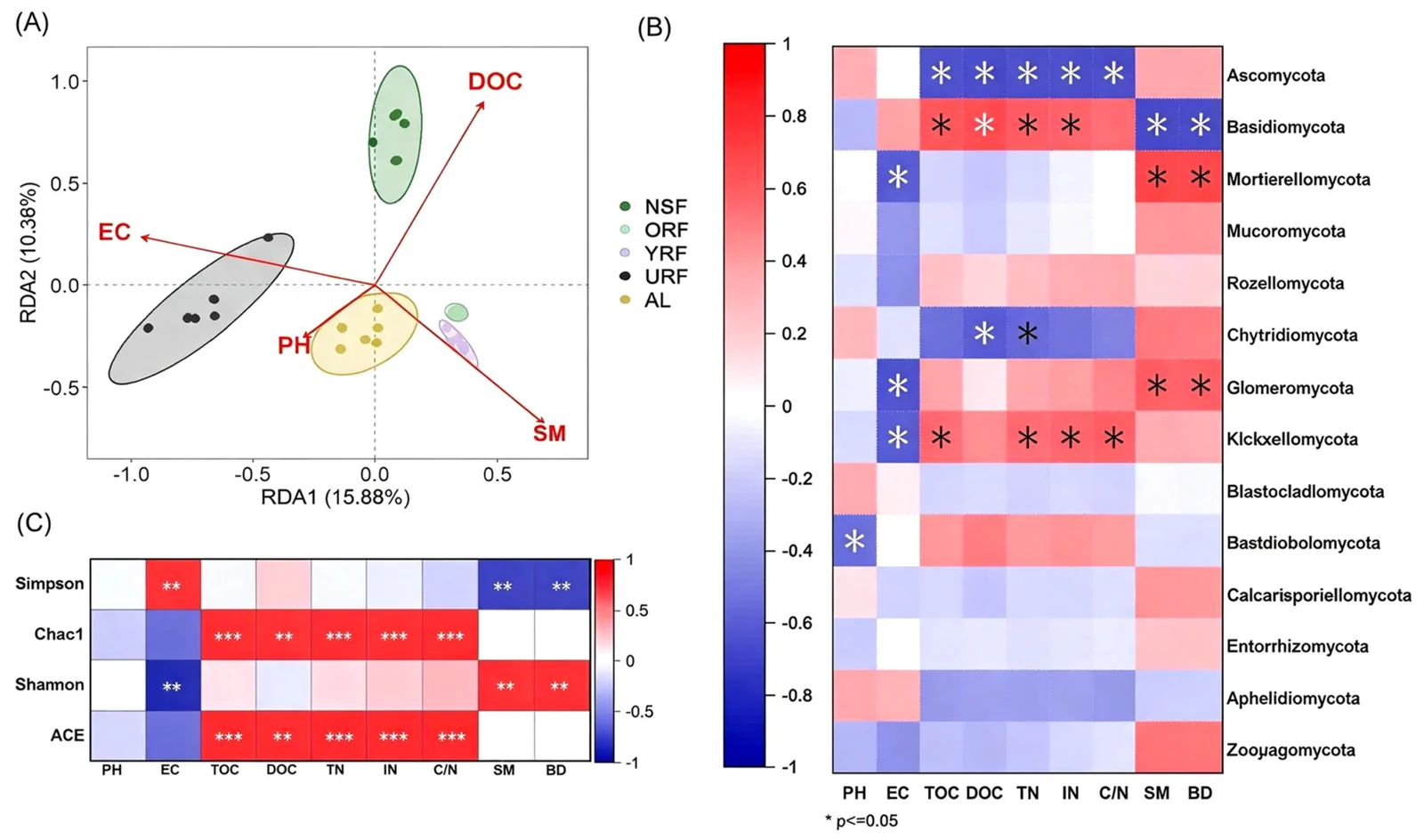
Relationship between soil physicochemical properties and soil fungal community composition and diversity indices. (**A**) Redundancy analysis (RDA) ordination of fungal communities constrained by soil properties. (**B**) Pearson correlation heatmap between dominant fungal phyla (relative abundance > 1%) and soil properties. (**C**) Pearson correlation heatmap between fungal diversity indices and soil properties. NSF, Natural Secondary Forest; ORF, Old Rubber Forest; YRF, Young Rubber Forest; URF, Upland Rice Field; AL, Abandoned Land. * *p* < 0.05, ** *p* < 0.01, *** *p* < 0.001. The color scale in panels (**B**,**C**) represents Spearman’s correlation coefficients, with red indicating positive correlations and blue indicating negative correlations.

RDA of soil fungal communities (Figure 6A) showed that the four retained soil physicochemical variables (pH, EC, DOC, SM) explained 26.24% of total variance, with RDA1 and RDA2 explaining 15.88 % and 10.36%, respectively. The global RDA model was statistically significant (F = 2.81, *p* = 0.001). Marginal permutation tests further confirmed that EC (F = 5.34, *p* = 0.001) and DOC (F = 3.51, *p* = 0.001) were the significant individual predictors shaping fungal community composition, whereas pH (F = 1.34, *p* = 0.122) and SM (F = 1.06, *p* = 0.321) showed non-significant effects. Vector fitting indicated DOC (R^2^ = 0.915, *p* < 0.001) had the strongest correlation with fungal community ordination, followed by EC (R^2^ = 0.881, *p* < 0.001) and SM (R^2^ = 0.868, *p* < 0.001), while pH showed no significant association (R^2^ = 0.137, *p* = 0.149). Pearson correlation analysis revealed differential relationships between soil properties and fungal phyla (Figure 6A,B). Ascomycota showed negative correlations with soil TOC, DOC, TN, and IN, whereas Basidiomycota showed positive correlations with these variables. Mortierellomycota and Glomeromycota showed positive correlations with SM and BD, but showed negative correlations with Basidiomycota. Soil properties were also correlated with fungal diversity (Figure 6C). DOC, TOC, TN, IN, and C/N were significantly correlated with Chao1 and ACE indices, while EC, SM, and BD were significantly correlated with Simpson and Shannon indices. Notably, no fungal diversity indices were significantly correlated with soil pH.

### 2.5. Predictive Analysis of Soil Microbial Functions

**Figure 7:**
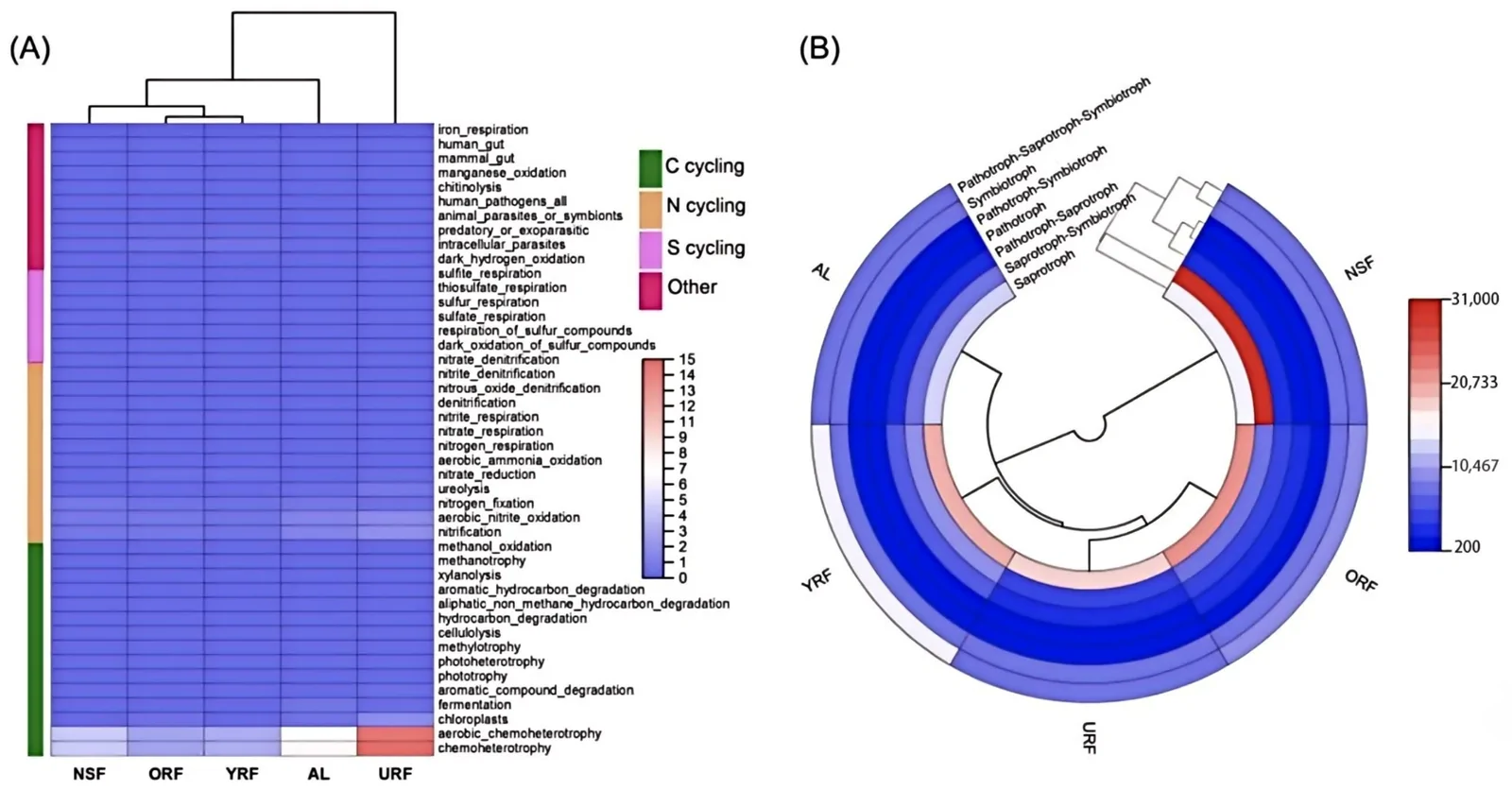
Relative abundance of soil bacterial functional groups (**A**) predicted by FAPROTAX and fungal trophic modes (**B**) predicted by FUNGuild. NSF, Natural Secondary Forest; ORF, Old Rubber Forest; YRF, Young Rubber Forest; URF, Upland Rice Field; AL, Abandoned Land. The color scale represents the relative abundance of soil bacterial functional groups and fungal trophic modes across land-use types, with red indicating higher abundance and blue indicating lower abundance.

FAPROTAX software (1.2.1) prediction identified 44 functional groups, which were predicted to be mainly associated with carbon and nitrogen cycling genes. C cycle functional groups included chemoheterotrophy, aerobic_chemoheterotrophy, chloroplasts, fermentation, aromatic_compound_degradation, phototrophy, photoheterotrophy, methylotrophy, cellulolysis, hydrocarbon_degradation, aliphatic_non_ methane_ hydrocarbon_degradation, aromatic_hydrocarbon_degradation, xylanolysis, methanotrophy, methanol_oxidation, etc. N cycle functional groups included nitrification, aerobic_nitrite_oxidation, nitrogen_fixation, ureolysis, nitrate_reduction, aerobic_ammonia_oxidation, nitrogen_respiration, nitrate_respiration, nitrite_respiration, denitrification, nitrous_oxide_denitrification, nitrite_denitrification, and nitrate_denitrification (Figure 7A), with aerobic_chemoheterotrophy (4.67–14.34%) and chemoheterotrophy (4.76–14.71%) being the highest in soil abundance in all five land-use types. The predicted relative abundance of functional groups involved in C, N, and S cycles varied among groups, with URF having the greatest relative abundance of C, N, and S cycle functional groups. The predicted relative abundance of C-cycling genes followed the order: URF > AL > NSF > YRF > ORF, whereas N-cycling genes followed the order: URF > AL > ORF > NSF > YRF.

FUNGuild software (1.0) was used to predict the ecological functions of soil fungi in different land use types. The results showed that the trophic modes of soil fungi under different land use types could be categorized into three types, namely, saprotroph, pathotroph, and symbiotroph (Figure 7B). Except for the symbiotroph and saprotroph trophic modes, the predicted relative abundance of other trophic modes varied significantly (*p* < 0.05) depending on the land-use types (Figure S1). For instance, the relative abundance of the saprotroph-symbiotroph trophic mode was significantly greater in NSF compared to all other land-use types (*p* < 0.05); conversely, the pathotroph-saprotroph trophic mode exhibited a lower predicted abundance in NSF, particularly when compared to YRF (*p* < 0.05). The pathotroph–saprotroph–symbiotroph trophic mode had significantly higher predicted abundance in YRF than in NSF and URF (*p* < 0.05), whereas no significant differences were detected between YRF and either ORF or AL. For the pathotroph trophic mode, its predicted abundance was significantly higher in AL relative to URF (*p* < 0.05); however, no significant variations were observed between AL and ORF, YRF, or NSF. For the pathotroph–symbiotroph trophic mode, its predicted abundance was significantly greater in NSF than in ORF (*p* < 0.05); additionally, ORF showed a significantly higher predicted abundance of this trophic mode compared to URF.

## 3. Discussion

### 3.1. Comparison of Soil Physicochemical Properties Along Two Land-Use Conversion Pathways

Land-use conversion from natural ecosystems to managed systems typically involves vegetation clearing and soil disturbance, leading to degradation reflected in altered physicochemical properties [34,35]. Previous studies have largely relied on either binary comparisons or chronosequence approaches within a single land-use type [19,20]. Even when multiple land-use types are compared, they are often confounded by variations in soil parent material, topography, or climate [21,22]. Neither approach adequately captures the stage-specific responses along successive land-use transitions from a common land-use background. In this study, our adjacent-plot design, with all sites sharing uniform parent material and climate, allowed us to detect the stage-specific responses along the successive land-use conversion in two pathways.

Overall, NSF exhibited the highest values for most soil properties. However, the two conversion pathways showed markedly different patterns. Along the NSF → ORF → YRF pathway, soil fertility indicators (TOC and TN) declined only mildly, consistent with previous reports that rubber replanting causes further but moderate soil degradation [36,37]. This is likely due to relatively stable canopy cover and limited soil disturbance, which slow organic matter and nutrient loss. In contrast, the NSF → ORF → URF pathway showed abrupt and severe degradation at the URF stage, where TOC and TN reached their lowest levels and EC peaked. Similar patterns of severe degradation following conversion to annual cropland have been observed in other tropical regions. For instance, forest-to-cropland conversion in Ethiopia cut organic matter by 48% and total nitrogen by 57% [38]; in the Philippines, peat swamp forest conversion to cropland caused above-ground carbon to decrease by 86–90% [39]. This pattern likely results from intensive farming, as tillage disrupts soil structure and accelerates decomposition, while excessive fertilization drives up salinity. These findings highlight the contrasting degradation trajectories between perennial and annual cropping systems following forest conversion.

It should be noted that the high soil quality observed in our secondary forest does not necessarily represent a universal pattern. Nazrin and Arifin (2025) found that in Malaysia, secondary forests had the lowest soil quality index among five land-use types, whereas mahogany plantations showed the highest [40]. Zhuo et al. (2026) reported that forest-to-orchard conversion in southern China actually increased soil nutrients [41]. These contrasting outcomes suggest that the ecological consequences of land-use conversion are highly context-dependent, shaped by site history, parent material, tree species, and management practices.

### 3.2. Divergent Responses of Bacterial and Fungal Communities to Land-Use Conversion

In this study, bacterial and fungal communities showed divergent response patterns. Bacterial diversity remained relatively stable across all conversion stages, with a significant change detected only in the Simpson index at the URF stage. In contrast, fungal diversity declined along the land-use conversion, particularly in the NSF → ORF → URF → AL pathway, and showed only partial recovery in AL, indicating cumulative sensitivity to vegetation and soil carbon changes, with very limited recovery even after abandonment [42]. These divergent responses between bacteria and fungi may be explained by fundamental ecological differences between the two groups. Bacterial stability is likely attributable to high functional redundancy, which allows multiple taxa to perform similar ecological roles and buffers the community against environmental fluctuations [43]; moreover, bacteria exhibit broad metabolic versatility and can rapidly colonize disturbed soils. By contrast, many fungi form intimate associations with plant roots as mycorrhizal symbionts and depend on specific litter inputs, rendering them more vulnerable to vegetation change [44]. These differences were further reflected at the genus level. Acidobacteria-affiliated genera (e.g., *Gp1*) dominated in NSF, ORF, and YRF but declined sharply in URF, whereas *Sphingomonas* and *Nitrospira* showed the opposite pattern (Table S3). Among fungi, ectomycorrhizal genera such as *Tomentella* and *Inocybe* nearly disappeared in converted lands, while *Mortierella* and *Fusarium* increased in AL or URF, respectively (Table S4). Therefore, the apparent stability of bacterial diversity belied substantial compositional shifts, reinforcing the role of functional redundancy in buffering community-level diversity against land-use conversion. Meanwhile, the sharp decline of symbionts alongside the rise in saprotrophic and pathogenic taxa underscores the sensitivity of fungal communities to vegetation and soil carbon changes.

The greater sensitivity of fungal communities observed in our study is consistent with previous reports [45,46]. Zhang et al. (2023) reported that soil fungal diversity was more sensitive than bacterial diversity to both mining disturbance and reforestation recovery in tropical rainforest ecosystems [45]. Kumar and Ghoshal (2017) further quantified this differential sensitivity, showing that fungal PLFAs explained 94% of the variability in soil organic carbon content compared to only 73% explained by bacteria [46]. However, in certain cases, bacteria can exhibit greater sensitivity than fungi. For instance, during natural afforestation of abandoned grasslands, bacterial communities were found to be more responsive to changes in soil properties, particularly pH, than fungal communities [47]. Similarly, under experimental nutrient enrichment, bacterial communities also exhibited stronger responses than fungi [48]. This differential sensitivity between microbial groups finds a parallel in observations in plant communities, where different functional types (e.g., drought avoiders vs. drought tolerators) exhibited divergent trait responses along soil water gradients, with trait divergence intensifying under more stressful conditions [23]. Collectively, these findings indicate that bacteria and fungi follow distinct ecological strategies (functional redundancy vs. vegetation sensitivity), shaping their divergent responses to land-use change and underscoring the need for taxon-specific assessments in evaluating tropical forest conversion.

### 3.3. Soil Physicochemical Properties Associated with Microbial Community Variation

Soil physicochemical properties drive microbial community structure, with their effects modulated by land-use conversion. Four soil variables (DOC, EC, WC, pH) were retained for RDA after VIF collinearity screening. The first two RDA axes explained 88.69% and 26.24% of constrained variation for bacterial and fungal communities, respectively, underscoring divergent responses of bacteria and fungi to soil physicochemical properties along the land-use conversion.

For bacteria, DOC showed the strongest independent effect on community composition, while EC and SM exhibited strong covariation with bacterial ordination space. DOC directly fuels microbial metabolism, and its concentration declined progressively from NSF to URF (0.231→0.057 g/kg), making it an increasingly limiting resource [49]. EC regulated bacteria primarily at URF, where salinity peaked (294.43 μS/cm) due to fertilization and irrigation [50]. Acidobacteria and Verrucomicrobia correlated positively with DOC, maintaining high abundance in low-intensity stages (NSF → ORF → YRF). In contrast, Proteobacteria thrived in URF and AL despite DOC depletion. This apparent contradiction likely reflects intensive management practices: frequent tillage and fertilization favor fast-growing, metabolically flexible taxa (r-strategists) that can rapidly exploit intermittent pulses of labile substrates derived from fresh organic matter inputs (e.g., crop residue incorporation and rhizo-deposition), even when the background DOC pool is depleted [51,52].

For fungal communities, DOC exhibited the strongest ordination association, followed by EC and SM; notably, only DOC and EC were statistically significant predictors of fungal community variation according to marginal tests. Consistently, Basidiomycota correlated positively with carbon variables, while Ascomycota correlated negatively, suggesting Ascomycota outcompete Basidiomycota under carbon limitation. EC shaped fungal communities and correlated with diversity indices, indicating salinity stress at URF. Basidiomycota declined substantially across all converted treatments compared with NSF (55.6%), yet URF remained relatively high (27.0%) despite the lowest TOC (4.35 g/kg), exceeding ORF (26.1%) and YRF (18.3%) (Figure 1). This indicates that carbon quantity alone did not drive its loss, as YRF showed the lowest abundance (18.3%) despite its higher TOC (7.45 g/kg), suggesting that non-carbon factors such as litter quality and tillage-induced hyphal disruption may have played a greater role [53,54,55]. The relatively small decline at URF may involve other non-carbon factors, warranting further verification. Mortierellomycota and Glomeromycota correlated positively with SM and BD, and were more abundant at AL, where abandonment alleviated compaction and restored moisture [56].

The divergent responses of bacteria and fungi observed here are consistent with broader patterns reported in other systems. Tian et al. (2012) demonstrated that land-use intensity alters DOC properties, which in turn shape microbial community structure [57]. Smith et al. (2014) further found that fungal-to-bacterial ratios decreased with decreasing aggregate size, indicating differential physical regulation of microbial groups [58]. More recently, Ribero et al. (2025) reported that grassland afforestation drove biotic homogenization of prokaryotes and fungi through distinct mechanisms [59]. Together with these previous observations, our findings reinforce that community-level changes are not determined by soil physicochemical properties alone, but are jointly regulated by disturbance intensity, substrate quality, and the inherent ecological traits of different microbial groups.

### 3.4. Predicted Microbial Functional Traits Under Different Land-Use Types

Microbial functional groups differed among the land-use types along the conversion pathway, reflecting the cumulative impact of increasing land-use intensity on soil ecological functions. For bacteria, FAPROTAX identified 44 functional groups, with chemoheterotrophy (4.76–14.71%) and aerobic chemoheterotrophy (4.67–14.34%) being the most abundant across all land-use types (Figure 7A). Both C- and N- cycling functional groups were most abundant at URF and AL, though their relative ranking of these groups varied across sites. Notably, C, N, and S cycling groups all peaked at URF despite the lowest TOC and DOC concentrations at this site (Table 1), suggesting that this functional enrichment results from accelerated nutrient turnover driven by tillage and fertilization, rather than from improved soil carbon status [60], consistent with Geng et al. (2026), who found that organic fertilization up-regulated nitrogen and phosphorus cycling genes in rubber plantations [61]. In contrast, along the NSF → ORF → YRF pathway, C-cycling functions declined with TOC and DOC decreases (Table 1), implying that carbon substrate limitation may constrain microbial functions in unfertilized rubber plantations [62].

For fungi, FUNGuild prediction revealed significant shifts in trophic modes along the conversion pathway. The saprotroph–symbiotroph mode, dominant in NSF, decreased progressively along the NSF → ORF → YRF → URF pathway, while the pathotroph–saprotroph mode increased, peaking at URF, where salinity stress (EC: 294.43 μS/cm) may have further selected for stress-tolerant pathotrophic taxa. This pattern is consistent with Brinkmann et al. (2019), who found that tropical forest conversion to rubber and oil palm plantations reduced symbiotrophs while increasing saprotrophs and pathotrophs [63], and with Liu et al. (2023), who reported similar shifts in Xishuangbanna rubber plantations [64]. At a broader scale, Banerjee et al. (2024) confirmed that agricultural intensification drives fungal homogenization and disproportionately affects symbiotic functional groups [65]. Overall, the shift from saprotroph–symbiotroph dominance in NSF to pathotroph–saprotroph dominance in URF suggests that high-intensity cultivation favors pathogenic fungi at the expense of symbiotic fungi, potentially compromising plant nutrient acquisition and stress tolerance, as previously observed in intensively managed tropical plantations [63,64].

It should be noted that functional predictions were based on FAPROTAX (for bacteria) and FUNGuild (for fungi), which rely on taxonomic reference databases and may not fully capture functional gene expression or intraspecific variation. Future studies should integrate metagenomics and targeted enzymatic assays for more definitive functional characterizations.

### 3.5. Early Signs of Amelioration in Abandoned Land and Its Limitations

In addition, this study found that AL, which had been abandoned for approximately six months, showed early signs of amelioration in some soil physicochemical properties, with values approaching those of YRF, although still far from NSF levels. This aligns with the broad consensus that natural succession plays a positive role in enhancing soil quality [24,25,26]. These initial improvements in soil properties in AL may be due to the restoration of vegetation cover, which reduces soil erosion and increases organic matter input through litter decomposition [66]. Functional groups also showed early signs of recovery. The predicted abundance of C, N, and S cycling functional groups in AL was lower than in URF but remained higher than in ORF and YRF, indicating a residual effect of prior agricultural disturbance that persisted after abandonment. In terms of fungal trophic modes, AL exhibited slight recovery of symbiotrophs and a reduction in pathotrophs compared to URF. Furthermore, fungal community composition in AL was closer to that observed in ORF and YRF than to the severely disturbed state at URF, indicating that six months of abandonment is sufficient to initiate fungal community recovery.

However, these improvements remain at an early stage. Sniegocki et al. (2022) reported that microbial diversity in Bornean tropical forests may require 60–80 years to fully recover to pre-disturbance levels, highlighting the prolonged timescale of fungal community recovery [42]. Pedrinho et al. (2019) similarly found that functional profiles in Amazonian secondary forests (13–15 years after pasture abandonment) only recovered to levels comparable to primary forest after more than a decade, further confirming the slow pace of recovery [67]. Thus, further recovery of AL is constrained by the short abandonment period (~0.5 years) and the persistent legacy effects of intensive agriculture, and full recovery to NSF levels may take decades or even longer. Synergizing natural recovery with active restoration has been demonstrated as a key pathway to accelerate the restoration of soil ecological functions [68]. Hence, for the ecological restoration of tropical degraded agricultural land, it is recommended to introduce timely active restoration measures on the basis of respecting natural succession.

## 4. Materials and Methods

### 4.1. Study Site

**Figure 8:**
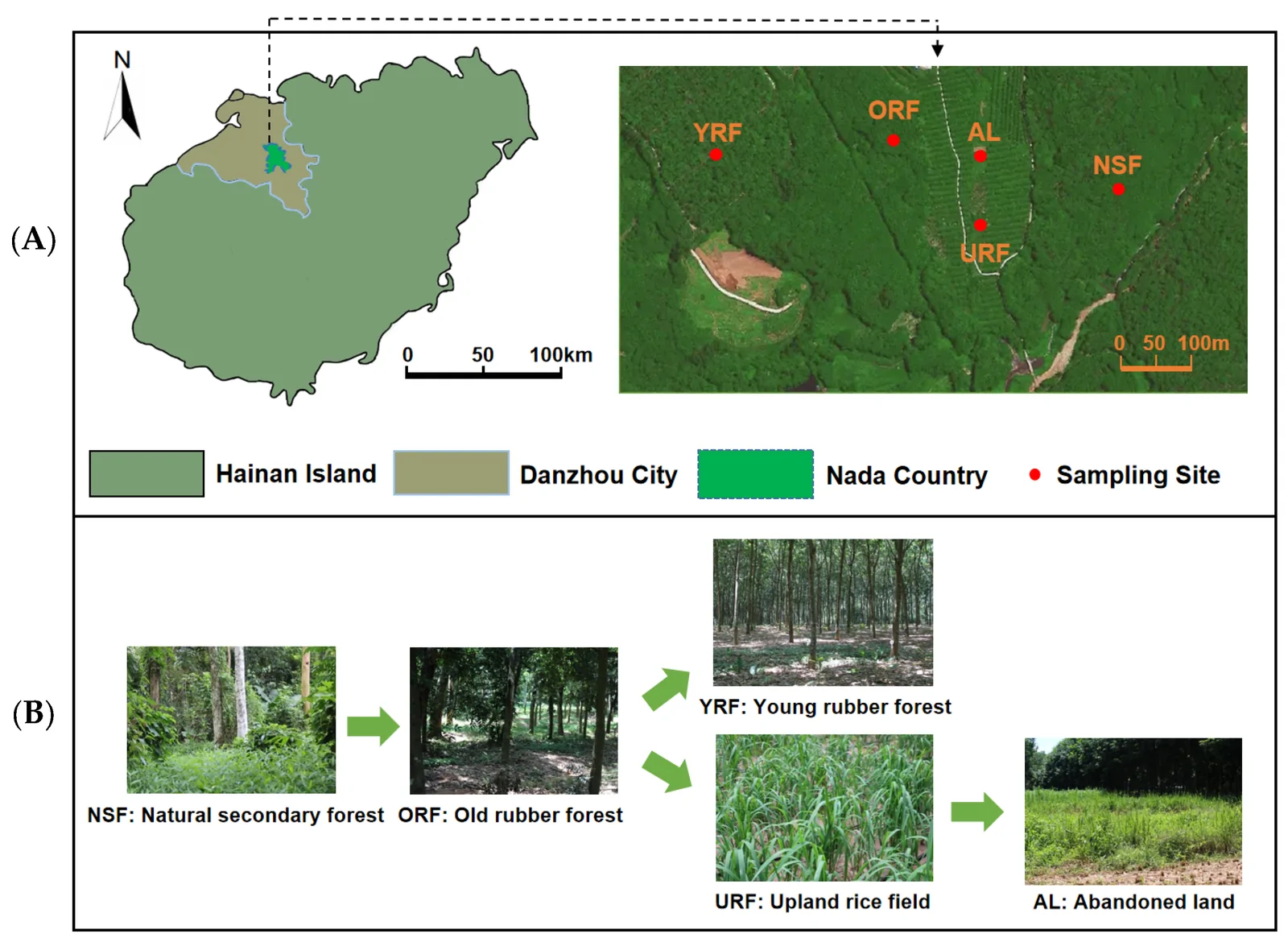
Schematic representation of sampling site locations (**A**) and land-use conversion pathways with representative photographs (**B**) at Liangyuan Experimental Agricultural Farm in Nada County, Danzhou City, Hainan Island, China. The five land-use types are: Natural Secondary Forest (NSF, >60 years), Old Rubber Forest (ORF, ~30 years), Young Rubber Forest (YRF, ~10 years), Upland Rice Field (URF, ~2 years), and Abandoned Land (AL, ~0.5 years). Green arrows indicate historical transitions between land-use types. All ages refer to the time since land-use establishment or last major disturbance up to the sampling date.

Soil samples for this study were gathered in August 2023 from Liangyuan Experimental Agricultural Farm (19°32′ N~19°34′ N, 109°28′ E~109°30′ E) in Nada Town, Danzhou City, Hainan Island, China (Figure 8A). The soils are deeply weathered Ferralsols, derived from granite. A thin organic layer (2–5 cm) is present in forested sites but is absent or minimal in cropland. The A horizon (0–20 cm) is loamy, overlying a clay loam B horizon (20–80 cm). Effective soil depth to weathered bedrock ranges from 80 to >150 cm. Surface soil (0–20 cm) properties across all land uses are: pH 5.52–5.77, TOC 4.35–14.24 g kg^−1^, TN 281.82–505.30 mg kg^−1^, and BD 1.22–1.47 g cm^−3^. The area experiences a typical tropical monsoon climate. Mean annual temperature ranges from 23 to 25 °C, with summer temperatures (May–October) around 26–32 °C and winter temperatures (November–February) around 16–22 °C. Annual precipitation ranges from 1500 to 2000 mm, with over 80% occurring in the rainy season (May–October), while the dry season (November–April) receives little rainfall and has abundant sunshine. The topography consists mainly of low hills and terraces at elevations of 80–120 m. Soil sampling was conducted during the rainy season, with no rainfall in the three days preceding collection to minimize transient moisture effects.

### 4.2. Soil Sampling

The fresh soil samples were collected from five land-use types within the same farm, with the maximum distance between any two sites being less than 500 m: Natural Secondary Forest (NSF, >60 years old), Old Rubber Forest (ORF, ~30 years old), Young Rubber Forest (YRF, ~10 years old), Upland Rice Field (URF, ~2 years old), and Abandoned Land (AL, ~0.5 years) (Figure 8B). All sites share the same soil parent material (granite-derived soils) and climatic conditions. This design minimizes confounding environmental variation and supports the validity of the space-for-time substitution approach. These land-use types constitute a land-use conversion sequence: ORF was converted from NSF, YRF was established after ORF clearing, URF was converted from former ORF stands, and AL corresponds to previously cultivated URF that was subsequently left fallow (Figure 8B). All reported ages represent the time elapsed since land-use establishment or the last major disturbance prior to soil sampling.

NSF is a multi-layered secondary forest dominated by native trees (e.g., *Antiaris toxicaria*, *Aphanamixis polystachya*), with diverse understory herbs and ferns, and no history of anthropogenic disturbance such as fertilization or tillage. ORF (~30 years) and YRF (~10 years) are rubber plantations (*Hevea brasiliensis*) established on former forestland, with an initial planting density of approximately 450–500 trees ha^−1^. Both stands received periodic weeding and fertilization during establishment, but weeding has since been discontinued in ORF, allowing shade-adapted understory recovery (e.g., *Dipteracanthus repens*, *Cyclosorus parasiticus*), while YRF remains under active management with a mixed understory of shade-tolerant and invasive herbs (e.g., *Ageratum conyzoides*, *Chromolaena odorata*). During rubber establishment, weeding was carried out twice a year, and fertilization was applied at a rate of 10 kg organic fertilizer plus 0.1 kg chemical fertilizer per tree. URF (~2 years) is an upland rice field converted from ORF. It is managed under conventional tillage (18–20 cm depth), with regular weeding and fertilization. Fertilizer application includes approximately 600 kg ha^−1^ compound fertilizer as basal, with urea topdressing at tillering, and drip irrigation is applied during dry spells. AL (~0.5 years) is the abandoned URF left fallow without any management, now dominated by ruderal grasses and forbs with no woody vegetation.

For each land-use type, six quadrats (10 m × 10 m) were randomly established, with a minimum distance of 20 m between adjacent quadrats. In each quadrat, five subsamples were randomly collected from the 0–20 cm depth (the most biologically active soil layer) using sterile shovels, following a systematic S-shaped sampling pattern. The five subsamples were pooled and homogenized into a single composite sample per quadrat, yielding a total of 30 composite samples (6 quadrats × 5 land-use types), which were treated as independent biological replicates for statistical analyses. After removal of stones, plant residues, and animal debris, each composite sample was divided into two aliquots: one was stored at −80 °C for DNA extraction and microbial analysis, and the other was air-dried in a cool, well-ventilated place, sieved through a 2 mm mesh, and stored at room temperature for determination of soil physicochemical properties. In this study, composite sampling averages within-quadrat heterogeneity and may obscure fine-scale spatial variation. However, this approach is appropriate for capturing representative conditions at the quadrat level and reducing the influence of localized anomalies.

### 4.3. Soil Properties

The soil physicochemical properties of each land use type were determined based on the mean values from six quadrat samples (10 m × 10 m) (Table 1). For physical properties, soil bulk density was determined by oven-drying soil samples in metal rings at 105 °C to constant weight, and soil moisture (SM) was calculated from the corresponding mass loss [21,69]. For chemical properties, the soil samples were air-dried at room temperature, sieved through a 2 mm mesh, and stored in airtight bags for subsequent analysis. Soil pH was measured using a PB-10 pH meter at a soil-to-water ratio of 1:5 (Sartorius GmbH, Göttingen, Germany) [70]. The pH meter was calibrated with standard buffer solutions before each measurement series. Soil electrical conductivity (EC) was measured from a 1:1 (soil:water) suspension using an EC meter (DDS-11A, Shanghai Leici Instrument Inc., Shanghai, China), which was calibrated using standard KCl solutions prior to measurements. Total organic carbon (TOC) was quantified by the K2Cr2O7 oxidation titration method [71]. Dissolved organic carbon (DOC) was measured using a total organic carbon analyzer (TOC-VWP, Shimadzu Corporation, Kyoto, Japan). Total nitrogen (TN) was determined using a fully automated Kjeldahl nitrogen tester (Kjeltec 8400, FOSS Analytical A/S, Hillerød, Denmark). Inorganic N (IN) in soils was measured using a UV-visible spectrophotometer (Model UV3600, Shimadzu, Japan). All measurements were performed in triplicate for each composite sample, and mean values were used for statistical analysis. Reagent blanks and standard reference materials (soil standard samples) were included in each batch of measurements to ensure analytical precision. The relative standard deviation (RSD) for replicated measurements was consistently below 5%. Finally, the soil carbon-to-nitrogen ratio (C/N) was determined as the ratio of soil total organic carbon to total nitrogen [72].

### 4.4. DNA Extraction and Sequencing of 16S rDNA and ITS

DNA was extracted from each composite soil sample using the E.Z.N.A™ Mag-Bind Soil DNA Kit (M5635-02, Omega, Norcross, GA, USA) following the manufacturer’s instructions. For each sample, approximately 0.5 g of soil was weighed into a 2 mL sterile tube. Cell disruption was performed using a bead-beating method: 0.1 g of sterile glass beads (0.1 mm diameter) and 0.3 g of zirconia/silica beads (0.5 mm diameter) were added to each tube, followed by vigorous shaking on a vortex mixer at maximum speed for 10 min to ensure efficient lysis of fungal cells and Gram-positive bacteria. The remaining extraction steps were carried out according to the manufacturer’s protocol. Genomic DNA integrity was examined by agarose gel electrophoresis, and genomic DNA concentrations were examined by a Qubit 4.0 spectrophotometer (ThermoFisher, Waltham, MA, USA). A negative control (sterile water) was included during DNA extraction and PCR amplification to monitor potential contamination. Primers 341F (5′-CCTACGGGNGGC WGCAG-3′) and 805R (5′-GACTACHVGGGTATCTAATCC-3′) were utilized to amplify the V3-V4 region of the 16S rRNA gene. Primers ITS1 (5′-CTTGTCATTTAGAGAAGTAA-3′) and ITS2 (5′- GTGCGT TCTTCATCGATGC-3′) were utilized to amplify the ITS1 highly variable region. The PCR amplification was conducted in a 30 μL reaction system containing 20 ng template DNA, 15 μL 2×Hieff^®^ Robust PCR Master Mix (Yeasen, Shanghai, China) and 1 μL of each primer. The two-step cycling protocol (5 initial cycles followed by 20 cycles) was designed to minimize non-specific amplification while ensuring sufficient product yield from low-biomass samples. The amplification reactions were conducted using a Thermal Cycler (Eastwin Life Sciences, Inc., Beijing, China) with the following conditions: denaturation at 94 °C for 5 min; followed by 5 cycles of 94 °C for 30 s, 45 °C for 20 s, and 65 °C for 30 s); then 20 cycles of 94 °C for 20 s, 55 °C for 20 s, and 72 °C for 30 s; with final extension at 72 °C for 5 min. The PCR products were detected by 2% agarose gel electrophoresis.

To ensure uniform cluster generation and high-quality sequencing data, library concentration was quantified using a Qubit^®^ 4.0 Fluorometer (ThermoFisher, Waltham, MA, USA). Finally, sequencing was performed on the Illumina MiSeq platform (Sangon, Shanghai, China). Raw reads were processed as follows: (1) adapter and primer sequences were trimmed using the cutadapt plugin, (2) the paired-end reads were merged using PEAR to generate consensus sequences, (3) samples were demultiplexed using sample-specific barcode and primer sequences, and (4) PRINSEQ was used for quality trimming, followed by the removal of sequences containing ambiguous bases (Ns), short reads (<200 bp), and low-complexity sequences. For bacterial sequences, a total of 2,461,182 high-quality reads (average 82,039 ± 9982 reads per sample) were obtained. For fungal sequences, a total of 2,815,271 high-quality reads (average 93,842 ± 13,423 reads per sample) were obtained. Good’s coverage exceeded 0.97 for all samples, indicating sufficient sequencing depth. Rarefaction curves approached saturation for all samples, confirming that the sequencing depth adequately captured the microbial diversity (Supplementary Figure S2). Sequences were clustered into OTUs at a 97% similarity level using USEARCH. Given the robustness of OTUs to sequencing errors at the moderate sequencing depth used here, we adopted OTU-based clustering at a 97% similarity threshold rather than exact ASVs. Taxonomic assignment of bacterial OTU representative sequences was performed using the RDP classifier (2.12) against the RDP database (http://rdp.cme.msu.edu/misc/resources.jsp) (1 September 2023) at a confidence threshold of 0.8, and taxonomic assignment of fungal OTU representative sequences was performed by a BLAST (2.10.0) search against the UNITE database (http://unite.ut.ee/index.php) (1 September 2023) at a confidence threshold of 0.6. Sequencing data have been deposited in the NCBI Sequence Read Archive (SRA) under accession number PRJNA1517184.

### 4.5. Statistical Analysis

All data were pre-processed using Excel 2019 and Origin 2025. To meet the assumptions of one-way ANOVA, normality (Shapiro–Wilk test) and homogeneity of variances (Levene’s test) were verified for all variables. Data that did not meet these assumptions were log-transformed or subjected to non-parametric tests. One-way ANOVA followed by Duncan’s multiple comparison test was performed using Origin 2025 software to evaluate significant differences in soil physicochemical properties and microbial diversity indices among different land-use types. Duncan’s test was chosen for pairwise comparisons among multiple groups, as it is appropriate for exploratory analyses to identify general trends in ecological datasets. Venn diagrams depicting shared and unique OTUs were used to illustrate the compositional overlap of soil bacterial and fungal communities across different land-use types, respectively. Soil microbial diversity indices, including the Chao1, ACE, Shannon, and Simpson indices, were calculated in Mothur based on OTU richness. To ensure comparability, all samples were rarefied to the same sequencing depth prior to diversity calculations. Bray–Curtis-based Principal Coordinate Analysis (PCoA) was used to compare the similarities and differences in microbial community composition among samples. To further assess the significance of community differentiation among land-use types, permutational multivariate analysis of variance (PERMANOVA) was performed based on Bray–Curtis distances with 9999 permutations.

Redundancy analysis (RDA) was used to analyze the correlations between soil physicochemical properties and microbial community composition. Prior to RDA analyses, low-abundance and spurious OTUs were filtered. Singletons and doubletons (OTUs with total reads ≤ 2) were removed. Prevalence was defined as the number of samples with non-zero reads; OTUs were retained when detected in ≥10% of samples for datasets with ≥20 samples, or in ≥3 samples for smaller datasets. Only OTUs with a mean relative abundance ≥ 0.01% (1 × 10^−4^) were kept. The filtered OTU matrix was Hellinger-transformed before redundancy analysis (RDA). Variance inflation factors (VIF) were calculated to test for multicollinearity among environmental variables; variables with VIF > 10 were excluded to control multicollinearity. Permutation tests with 999 permutations were conducted to test the global significance of the RDA model and marginal effects of individual retained variables. Vector fitting analysis (envfit, 999 permutations) was applied to quantify correlations between environmental variables and the RDA ordination space for plotting. Correlations between soil physical and chemical properties and soil microbial communities at the phylum level as well as with soil microbial diversity indices were analyzed based on Spearman correlation analysis, and taxa significantly correlated with each environmental factor were screened and visualized using correlation heat maps. For the multiple Spearman correlation tests, the Benjamini–Hochberg false discovery rate (FDR) correction was applied to control the type I error rate. The ecological functions of bacteria and fungi were annotated using FAPROTAX and FUNGuild tools [73,74], respectively. While these tools provide valuable functional predictions, we acknowledge their limitations, including incomplete database coverage and potential inaccuracies in functional assignment for understudied taxa. All data were visualized using R software (4.4.3) or Origin 2025 software.

## 5. Conclusions

This study of successive land-use transitions revealed stage-specific responses of soil properties and microbial communities to tropical land-use conversion, providing insights that are unattainable through binary comparisons. NSF exhibited optimal soil fertility and microbial structure. The NSF → ORF → YRF pathway caused gradual, mild degradation, while the NSF → ORF → URF pathway led to abrupt, severe degradation at URF, with AL achieving only partial recovery at this early stage (~0.5 years of abandonment). Under the successive land-use conversions, fungi were more sensitive than bacteria, with diversity declining progressively and dominant phyla shifting from Basidiomycota to Ascomycota, positioning fungi as more effective bioindicators than bacteria. DOC, EC, and SM were identified as the variables most strongly associated with microbial community ordination, whereas pH showed no significant association. URF showed C/N/S cycling functions, while NSF maintained a balanced fungal trophic structure. Notably, recovery in AL did not reverse the degradation path: fungal communities in AL clustered with those in ORF and YRF rather than NSF, revealing a clear path-dependent trajectory. This study contributes to a shift from static comparisons to a dynamic, stage-specific understanding of land-use change impacts. Based on these findings, we propose stage-specific management recommendations: NSF as a priority protection area; ORF with optimized management to prevent further degradation; YRF with enhanced organic inputs; URF requiring emergency intervention through cessation of intensive practices and initiation of assisted restoration; and AL supporting natural succession with targeted amendments. However, these findings should be interpreted with caution, as this study was conducted at a single site with one-time sampling during the rainy season, and how soil properties and microbial communities respond during the dry season remains unknown. AL represents only an early stage (~0.5 years) of succession, and functional predictions are inferred from sequence data rather than directly measured, warranting confirmation through metagenomic or metatranscriptomic approaches in future studies.

## Figures and Tables

**Table 1:** Characterization of soil physicochemical properties in different land use types (0–20 cm).

Soil Properties		NSF	ORF	YRF	URF	AL
Chemical properties	pH	5.52 ± 0.30 a	5.68 ± 0.20 a	5.60 ± 0.38 a	5.77 ± 0.19 a	5.65 ± 0.16 a
	EC (μS/cm)	237.97 ± 6.65 b	194.87 ± 12.27 c	198.37 ± 14.76 c	294.43 ± 21.00 a	211.97 ± 15.28 bc
	TOC (g/kg)	14.24 ± 1.81 a	9.70 ± 1.35 b	7.45 ± 0.20 c	4.35 ± 0.57 d	7.62 ± 0.57 c
	DOC (g/kg)	0.231 ± 0.008 a	0.132 ± 0.002 b	0.096 ± 0.002 c	0.057 ± 0.001 d	0.098 ± 0.002 c
	TN (mg/kg)	505.30 ± 43.06 a	403.88 ± 28.04 b	351.15 ± 2.95 c	281.82 ± 11.80 d	355.85 ± 14.24 c
	IN (mg/kg)	7.60 ± 0.60 a	6.21 ± 0.31 b	5.47 ± 0.09 c	4.26 ± 0.13 d	5.58 ± 0.20 c
	C/N	28.11 ± 1.17 a	23.95 ± 1.62 b	21.22 ± 0.45 c	15.40 ± 1.43 d	21.40 ± 0.76 c
Physical properties	BD (g/cm^3^)	1.22 ± 0.06 b	1.45 ± 0.07 a	1.47 ± 0.09 a	1.23 ± 0.06 b	1.40 ± 0.07 a
	SM (%)	20.03 ± 3.78 a	21.17 ± 1.94 a	19.08 ± 1.78 a	12.45 ± 0.52 b	18.83 ± 1.57 a

Note: EC, electrical conductivity; TOC, total organic carbon; DOC, dissolved organic carbon; TN, total nitrogen; IN, inorganic N; C/N: the ratio of TOC to TN; BD: bulk density; SM: soil moisture. Values are mean ± SD (*n* = 6). Different lowercase letters indicate statistically significant differences among land use types (*p* < 0.05, Duncan’s multiple range test). NSF, Natural Secondary Forest; ORF, Old Rubber Forest; YRF, Young Rubber Forest; URF, Upland Rice Field; AL, Abandoned Land.

## Data Availability

The original contributions presented in this study are included in the article/Supplementary Materials. Further inquiries can be directed to the corresponding authors.

## Supplementary Materials

The following supporting information can be downloaded at: https://www.mdpi.com/article/10.3390/plants15203091/s1. Table S1. Relative abundance of soil bacterial phyla across five land-use types. Table S2. Relative abundance of soil fungal phyla across five land-use types. Table S3. Relative abundance of soil bacterial genera across five land-use types. Table S4. Relative abundance of soil fungal genus across five land-use types. Figure S1. Differences in the relative abundance of soil fungal trophic modes among different land-use types. Figure S2. Rarefaction curves of bacterial communities based on 16S rRNA (A) and ITS (B) gene sequencing.
