# Supplementary material for: Impacts of Two Land-Use Conversion Pathways on Soil Properties and Microbial Communities in Tropical Forests of Hainan Island
Source: Plants (Basel). 2026 Oct 9;15(20):3091. doi: 10.3390/plants15203091 (PMC13709285; doi:10.3390/plants15203091)
Supplement: Supplementary file 1 [file plants-15-03091-s001.zip › Figure S1 .pdf]

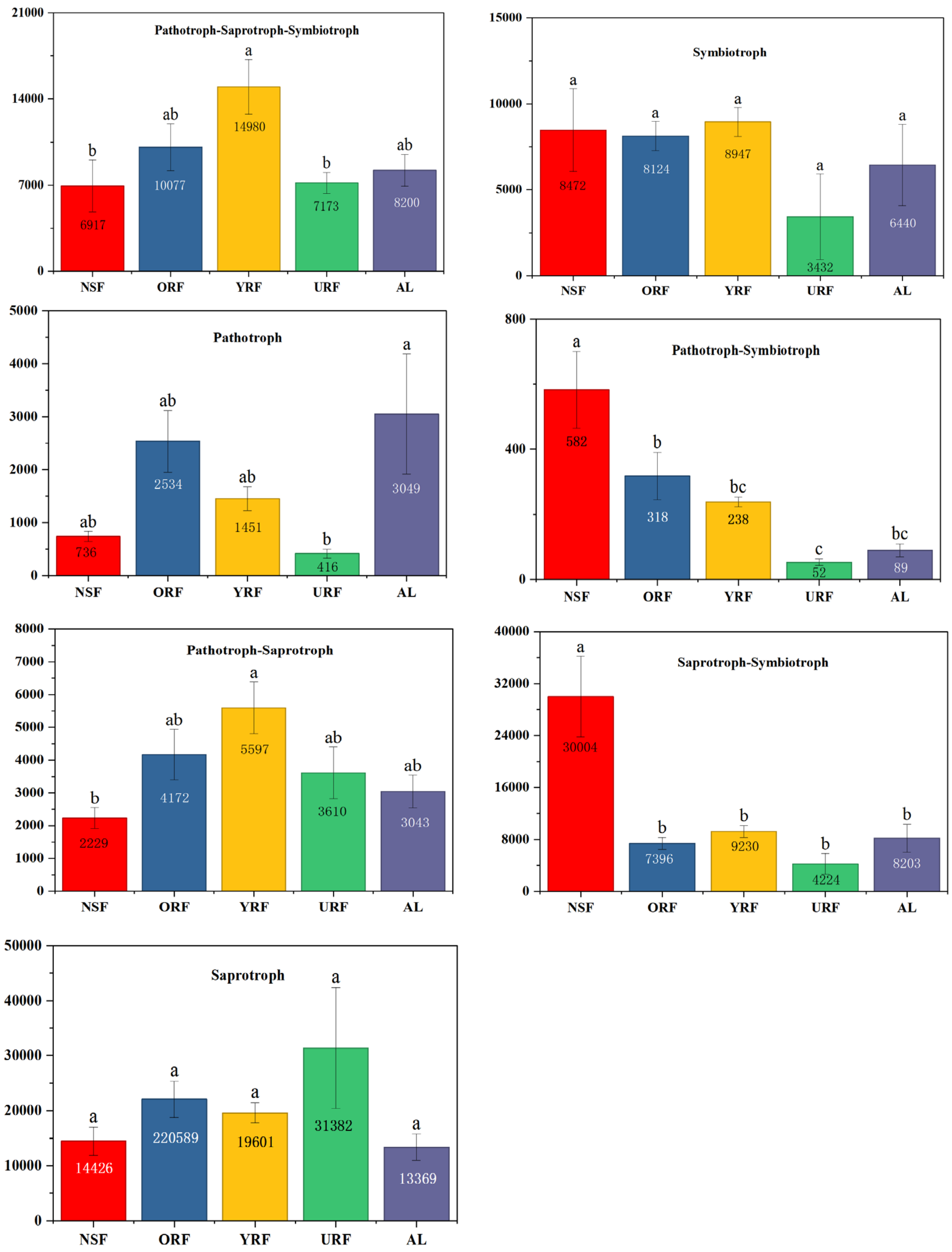

Figure S1. Differences in the relative abundance of soil fungal trophic modes among different land-use types. NSF, ORF, YRF, URF, and AL represent Natural Secondary Forest, Old Rubber Forest, Young Rubber Forest, Abandoned Land, and Upland Rice Field, respectively.
