# Supplementary material for: Impacts of Two Land-Use Conversion Pathways on Soil Properties and Microbial Communities in Tropical Forests of Hainan Island
Source: Plants (Basel). 2026 Oct 9;15(20):3091. doi: 10.3390/plants15203091 (PMC13709285; doi:10.3390/plants15203091)
Supplement: Supplementary file 1 [file plants-15-03091-s001.zip › Table S1.pdf]

Table S1. Relative abundance of soil bacterial phyla across five land-use types.NSF, ORF, YRF, URF, and AL represent Natural Secondary Forest, Old Rubber Forest, Young Rubber Forest, Abandoned Land, and Upland Rice Field, respectively.

Number	phylum	NSF	ORF	YRF	URF	AL
1	Acidobacteria	35.300	36.879	37.504	18.741	25.036
2	Proteobacteria	31.569	25.404	26.342	35.459	33.348
3	Verrucomicrobia	7.672	4.341	4.905	2.999	3.082
4	Actinobacteria	7.968	4.698	6.052	8.617	7.182
5	Bacteroidetes	2.177	2.837	1.873	2.703	2.586
6	Firmicutes	1.035	0.978	0.504	2.536	2.140
7	Gemmatimonadetes	1.759	1.127	1.555	4.071	2.723
8	Planctomycetes	1.781	2.185	2.104	2.405	2.378
9	Chloroflexi	0.726	4.066	3.312	2.759	3.785
10	candidate_division_WPS-1	1.281	0.759	0.810	2.050	1.891
11	Nitrospirae	0.752	0.865	0.582	2.288	1.395
12	candidate_division_WPS-2	0.918	0.504	1.009	1.196	0.662
13	Cyanobacteria_Chloroplast	0.133	0.473	0.070	2.360	0.807
14	Candidatus_Saccharibacteria	0.400	0.318	0.413	1.158	0.795
15	unclassified_Bacteria	6.198	13.608	11.991	9.941	11.497
16	Other	0.330	0.959	0.973	0.717	0.693
