# Supplementary material for: Impacts of Two Land-Use Conversion Pathways on Soil Properties and Microbial Communities in Tropical Forests of Hainan Island
Source: Plants (Basel). 2026 Oct 9;15(20):3091. doi: 10.3390/plants15203091 (PMC13709285; doi:10.3390/plants15203091)
Supplement: Supplementary file 1 [file plants-15-03091-s001.zip › Table S2.pdf]

Table S2. Relative abundance of soil fungal phyla across five land-use types. NSF, ORF, YRF, URF, and AL represent Natural Secondary Forest, Old Rubber Forest, Young Rubber Forest, Abandoned Land, and Upland Rice Field, respectively.

Number	phylum	NSF	ORF	YRF	URF	AL
1	Ascomycota	33.276	51.971	59.805	63.991	46.531
2	Basidiomycota	55.646	26.097	18.273	27.024	22.251
3	Mortierellomycota	2.477	8.476	7.801	2.192	8.894
4	Mucoromycota	0.817	1.403	2.482	0.226	7.317
5	Rozellomycota	3.432	2.819	2.681	0.934	6.272
6	Chytridiomycota	0.752	2.109	3.328	2.790	2.772
7	Glomeromycota	1.693	4.630	3.063	0.255	2.530
8	Kickxellomycota	0.062	0.109	0.029	0.015	0.054
9	Blastocladiomycota	0.055	0.220	0.024	0.223	0.045
10	Basidiobolomycota	0.028	0.008	0.002	0.008	0.005
11	Calcarisporiellomycota	0.009	0.095	0.035	0.041	0.005
12	Entorrhizomycota	0.000	0.000	0.000	0.000	0.002
13	Aphelidiomycota	0.000	0.001	0.000	0.003	0.001
14	Zoopagomycota	0.000	0.002	0.011	0.000	0.009
15	unclassified_Fungi	1.755	2.059	2.464	2.300	3.313
