# Supplementary material for: Impacts of Two Land-Use Conversion Pathways on Soil Properties and Microbial Communities in Tropical Forests of Hainan Island
Source: Plants (Basel). 2026 Oct 9;15(20):3091. doi: 10.3390/plants15203091 (PMC13709285; doi:10.3390/plants15203091)
Supplement: Supplementary file 1 [file plants-15-03091-s001.zip › Table S3.pdf]

Table S3. Relative abundance of soil bacterial genera across five land-use types.NSF, ORF, YRF, URF, and AL represent Natural Secondary Forest, Old Rubber Forest, Young Rubber Forest, Abandoned Land, and Upland Rice Field, respectively.

Number	genera	NSF	ORF	YRF	URF	AL
1	*Gp1*	12.201	9.001	13.039	6.079	10.822
2	*Sphingomonas*	0.687	0.300	0.471	7.640	2.725
3	*Acidibacter*	2.530	1.337	1.818	0.664	1.467
4	*Nitrospira*	0.752	0.865	0.582	2.288	1.395
5	*Gaiella*	1.693	0.650	0.792	2.608	2.045
6	*Edaphobacter*	1.316	0.312	0.593	0.155	0.372
7	unclassified_Rhizobiales	3.286	1.477	1.418	1.621	2.161
8	Other	77.535	86.058	81.287	78.946	79.014
