# Supplementary material for: Impacts of Two Land-Use Conversion Pathways on Soil Properties and Microbial Communities in Tropical Forests of Hainan Island
Source: Plants (Basel). 2026 Oct 9;15(20):3091. doi: 10.3390/plants15203091 (PMC13709285; doi:10.3390/plants15203091)
Supplement: Supplementary file 1 [file plants-15-03091-s001.zip › Table S4.pdf]

**Table S4.** Relative abundance of soil fungal genus across five land-use types. NSF, ORF, YRF, URF, and AL represent Natural Secondary Forest, Old Rubber Forest, Young Rubber Forest, Abandoned Land, and Upland Rice Field, respectively.

| Number | genus | NSF | ORF | YRF | URF | AL |
|---|---|---|---|---|---|---|
| 1 | *Tomentella* | 26.804 | 0.872 | 0.774 | 2.002 | 0.686 |
| 2 | *Inocybe* | 8.616 | 0.212 | 0.176 | 0.134 | 0.177 |
| 3 | *Scleroderma* | 3.456 | 0.220 | 0.161 | 2.666 | 0.166 |
| 4 | *Mortierella* | 2.093 | 6.712 | 6.656 | 1.960 | 8.576 |
| 5 | *Fusarium* | 1.073 | 1.087 | 1.875 | 4.962 | 7.879 |
| 6 | *Conocybe* | 0.169 | 1.011 | 0.173 | 0.095 | 7.015 |
| 7 | *Rhizoctonia* | 0.288 | 0.165 | 0.157 | 0.046 | 2.782 |
| 8 | Other | 57.502 | 89.720 | 90.028 | 88.136 | 72.720 |
